# Occurrence and Toxicogenetic Profiling of *Clostridium perfringens* in Buffalo and Cattle: An Update from Pakistan

**DOI:** 10.3390/toxins13030212

**Published:** 2021-03-13

**Authors:** Muhammad Umar Zafar Khan, Muhammad Humza, Shunli Yang, Mughees Aizaz Alvi, Muhammad Zahid Iqbal, Hafiza Zain-ul-Fatima, Shumaila Khalid, Tahir Munir, Jianping Cai

**Affiliations:** 1 State Key Laboratory of Veterinary Etological Biology, Key Laboratory of Veterinary Parasitology of Gansu Province, Lanzhou Veterinary Research Institute, Chinese Academy of Agricultural Sciences, Lanzhou 730046, China; umarzafar8@caas.cn (M.U.Z.K.); yangshunli@caas.cn (S.Y.); 2 Jiangsu Co-Innovation Center for the Prevention and Control of Important Animal Infectious Disease and Zoonoses, Yangzhou University, Yangzhou 225009, China; 3 Key Laboratory of Agro-products Quality and Safety Control in Storage and Transport Process, Ministry of Agriculture and Rural Affairs/ Institute of Food Science and Technology, Chinese Academy of Agricultural Sciences, Beijing 100193, China; 2017Y90100131@caas.cn; 4 Department of Plant Pathology, University of Agriculture, Faisalabad 38000, Pakistan; 5 Department of Clinical Medicine and Surgery, University of Agriculture, Faisalabad 38000, Pakistan; mugheesaizazalvi@gmail.com; 6 Department of Veterinary Medicine, University of Veterinary and Animal Sciences, Outfall Road, Lahore 54000, Pakistan; zahid.iqbal@uvas.edu.pk; 7 Veterinary Research Institute, Zarrar Shaheed Road, Lahore Cantt., Lahore 54810, Pakistan; dr.hafiza.zain@gmail.com; 8 Department of Livestock and Dairy Development, Lahore 54000, Pakistan; shumailakhalid.sk@gmail.com (S.K.); dr.tahir99@gmail.com (T.M.)

**Keywords:** *Clostridium perfringens*, toxins, genotypes, occurrence, optimization, This manuscript states the occurrence of toxinotypes isolated from *Clostridium perfringens* in bovine areas of Punjab province in Pakistan and it can be applied to the proper prediction of the isolated toxinotypes. The genotyping could be helpful in appropriate diagnosis.

## Abstract

*Clostridium perfringens* is a Gram-positive bacterium that possess seven toxinotypes (A, B, C, D, E, F, and G) that are responsible for the production of six major toxins, i.e., α, β, ε, ι, *CPE*, and *NetB*. The aim of this study is to find out the occurrence of toxinotypes in buffalo and cattle of Punjab province in Pakistan and their corresponding toxin-encoding genes from the isolated toxinotypes. To accomplish this aim, six districts in Punjab province were selected (i.e., Lahore, Sahiwal, Cheecha Watni, Bhakkar, Dera Ghazi Khan, and Bahawalpur) and a total of 240 buffalo and 240 cattle were selected for the collection of samples. From isolation and molecular analysis (16S rRNA), it was observed that out of seven toxinotypes (A–G), two toxinotypes (A and D) were found at most, whereas other toxinotypes, i.e., B, C, E, F, and G, were not found. The most frequently occurring toxinotype was type A (buffalo: 149/240; cattle: 157/240) whereas type D (buffalo: 8/240 cattle: 7/240) was found to occur the least. Genes encoding toxinotypes A and D were *cpa* and *etx*, respectively, whereas genes encoding other toxinotypes were not observed. The occurrence of isolated toxinotypes was studied using response surface methodology, which suggested a considerable occurrence of the isolated toxinotypes (A and D) in both buffalo and cattle. Association between type A and type D was found to be significant among the isolated toxinotypes in both buffalo and cattle (*p* ≤ 0.05). Correlation was also found to be positive and significant between type A and type D. *C. perfringens* exhibits a range of toxinotypes that can be diagnosed via genotyping, which is more reliable than classical toxinotyping.

## 1. Introduction

The livestock industry is one of the fastest-growing and most financially important sub-sectors of the economy in developing countries such as Pakistan, accounting for a 60.54% value addition to the Pakistani agricultural sector and contributing 11.22% to the GDP (gross domestic product). Furthermore, it makes a significant contribution to rural economic growth, where 40% of the population is dependent on livestock. Unfortunately, over the last decade, the bovine industry has significantly declined due to infectious diseases [1].

*Clostridium perfringens* is well known for its ability to express a wide variety of exotoxins/enzymes. The pathogenesis of *C. perfringens* diseases is directly associated with its prolific toxin-producing ability [2,3,4]. The epsilon toxin (ETX) is considered the most potent toxin produced by this bacteria and it has been also considered a category B toxin by the Centers for Disease Control (CDC) for its potential use in bioterrorism [5,6]. This toxin is produced by *C. perfringens* types B and D as a prototoxin, activated through proteolytic cleavage by the animal’s trypsin, quimotrypsin, or *C. perfringens* zinc-metalloprotease and then absorbed into the blood stream, affecting other organs such as the brain [6,7]. The pathogenic clostridial species can be classified into three groups based on their toxin activity (enterotoxic, histotoxic, and neurotoxic) on target tissues [8]. *C. perfringens* type A has been and is still frequently blamed for enteritis, abomasitis, and/or enterotoxaemia in cattle [9]. The coding gene (CPA) is highly conserved and thus present in all strains of *C. perfringens* [10]. CPA plays a critical role in the pathogenesis of gas gangrene in humans and animals [11,12]. However, in recent years, multiple lines of evidence from several well-designed studies mainly based on molecular Koch’s postulates demonstrated the critical role of CPA in the pathogenesis of bovine necro-hemorrhagic enteritis [13,14,15]. *C. perfringens* toxinotype D isolates tend to produce alpha and epsilon toxins, which are encoded by the plc and etx genes, leading to the development of enterotoxaemia (“pulpy kidney”) in sheep and goats [16,17,18]. *C. perfringens* type D (epsilon toxin) is responsible for enterotoxaemia in sheep, goats, and cattle [19].

Although *C. perfringens* can exist as part of the normal intestinal microflora, under certain conditions where the physiological balance of the intestine is altered, abnormal proliferation of this microorganism can result in the production of a wide variety of toxins. There are very few reports on the toxin profile of *C. perfringens* isolates recovered from buffalo and cattle in Pakistan. More comprehensive information about *C. perfringens* types A and D in these animals is also lacking. As such, the aim of this study is to determine the occurrence of major toxinotypes and their corresponding toxins produced by *C. perfringens* in buffalo and cattle. Furthermore, a molecular characterization was carried out to find out the toxin-encoding genes causing enterotoxaemia in bovines (buffalo and cattle). The statistical optimization would predict the possible occurrence of the isolated toxinotypes (A and D) in buffalo and cattle. This approach could serve as a predictor for the determination of possible occurrence in the bovine population of Punjab province, Pakistan.

## 2. Results

### 2.1. Geographical Distribution of C. perfringens Toxinotypes in Sampled Areas of Punjab Province in Pakistan

**Figure 1:**
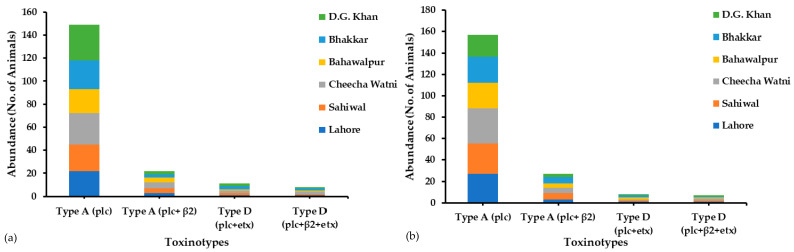
Abundance of *C. perfringens* toxinotypes in (**a**) buffalo and (**b**) cattle samples collected from Central and Southern Punjab province, Pakistan.

During the sample collection from six districts of Punjab province, Pakistan, we surveyed various farms and household animals for the occurrence of all *C. perfringens* toxinotypes (A–G). The most prevalent toxin genotypes were type A (plc) and type A (plc + β2), as most of the infected animals showed both of these genotypes at a maximum rate. In Central Punjab (Lahore, Sahiwal, and Cheecha Watni), 60% of the sampled buffalo and 73% of the sampled cattle and in Southern Punjab (Bhakkar, Dera Ghazi Khan, and Bahawalpur), 64% of sampled buffalo and 57% of the sampled cattle showed the type A (plc) genotype. In total, 149 buffalo out of 240 and 22 buffalo out of 240 showed Type A (plc + etx) and Type A (plc + β2) genotypes, respectively, whereas 157 cattle out of 240 and 21 cattle out of 240 showed type A (plc) and type A (plc + β2) genotypes, respectively, in both Central and Southern Punjab. Type D (plc + β2 + etx) showed less abundance in both buffalo and cattle (Table 1). The occurrence of these toxinotypes in both regions is illustrated in Figure 1, in which each site has its own abundance of toxinotypes of *C. perfringens* origin. Conclusively, toxinotype A was found more compared to toxinotype D. Other toxinotypes (B, C, E, F, and G) were not observed in either buffalo or cattle.

### 2.2. Isolation of Toxinotypes Isolated from C. perfringens in Buffalo and Cattle

**Figure 2:**
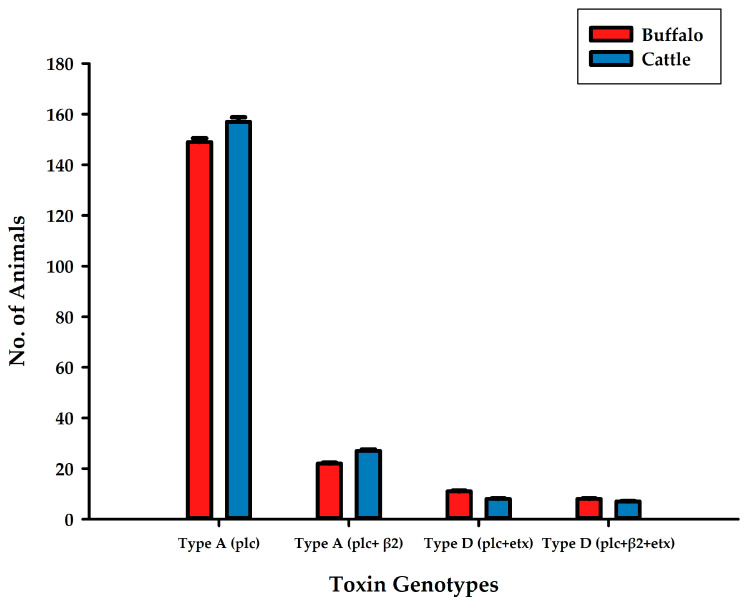
Availability of toxin genotypes A (plc), A (plc + β2), D (plc + etx), D (plc + β2 + etx) isolated from *C. perfringens* in buffalo and cattle samples from different cities in Central and Southern Punjab province, Pakistan.

After the isolation of *C. perfringens* and identification of toxin genotypes in both buffalo and cattle, type A (plc) showed maximum incidence in both buffalo and cattle, as all the examined animals were found prone to this genotype (Figure 2). Type D (plc + β2 + etx) was found to be minimum in buffalo and cattle. Type A-based toxinotypes were found more compared to type D-based toxinotypes.

### 2.3. Principal Component Analysis of Toxinotypes in Buffalo and Cattle

The principal component analysis was performed to find out the relationship among the toxinotypes of *C. perfringens* in buffalo and cattle. According to this analysis, a PCA plot was for both Buffalo and cattle separately.

**Figure 3:**
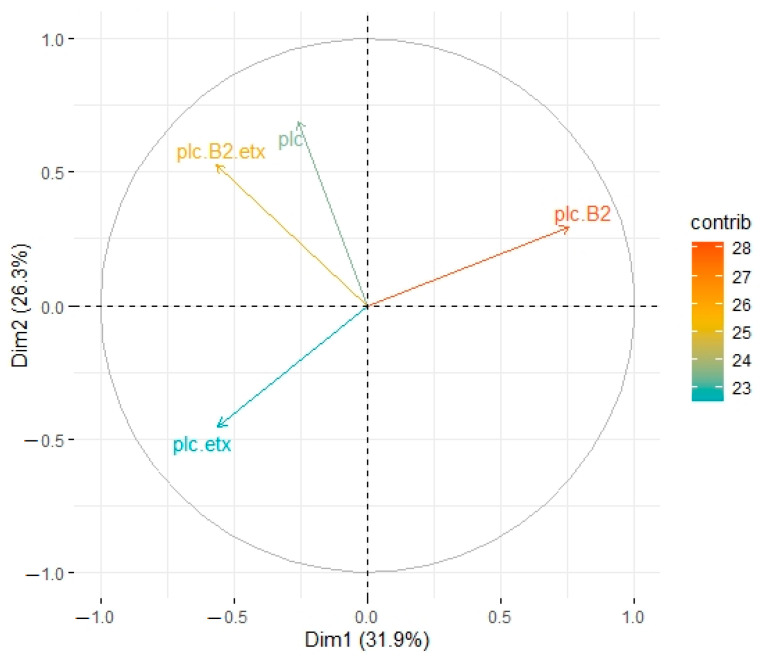
Principle component analysis of toxinotypes isolated from *C. perfringens* in buffalo. Type A (plc) is represented by a blue line, type A (plc + β2) is represented by an orange line, type D (plc + etx) is represented by a red line, and type D (plc + β2 + etx) is represented by a green line.

In buffalo, both principal components PC1and PC2 accounted for 32% and 26% variation among the toxinotypes of bacterial origin, respectively. Genotype A (plc) exhibited significant association with all other genotypes except type D (plc + etx), whereas a significant association was observed in the case of other genotypes among each other (*p* ≤ 0.05). Type A (plc + β2) and type D (plc + β2 + etx) showed slightly non-significant association and type D (plc + β2 + etx) also showed non-significant association with type A (plc + β2) but significant association was observed with type D (plc + etx). Type A (plc + β2) and (plc + β2) showed maximum response compared to genotype D (plc + etx) in contribution level (Figure 3).

**Figure 4:**
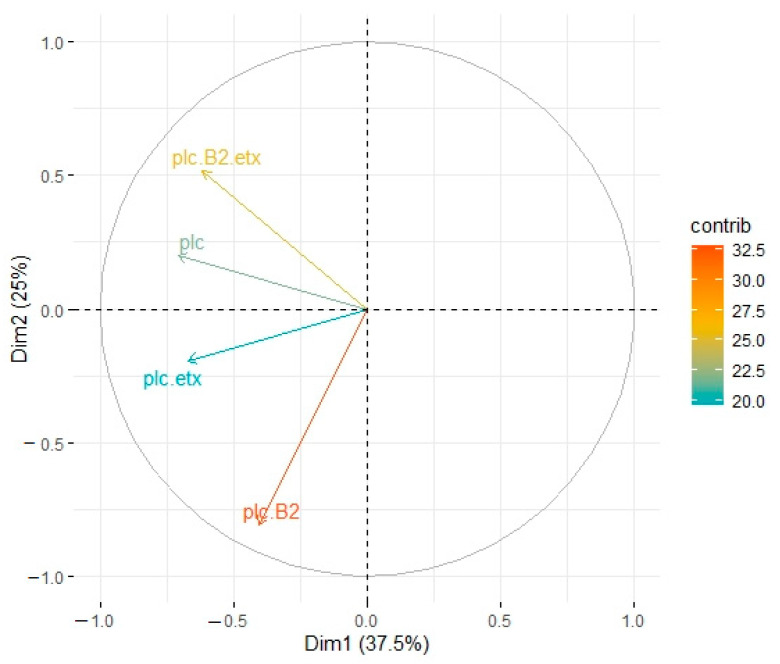
Principle component analysis of toxinotypes isolated from *C. perfringens* in cattle. Type A (plc) is represented by a blue line, type A (plc + β2) is represented by a blue line, type D (plc + etx) is represented by a red line, and type D (plc + β2 + etx) is represented by a green line.

In cattle, PC1 accounted for 37% whereas PC2 accounted for 25% of variation among different genotypes (Figure 4). Genotype A (plc + β2) displayed significant association with type A (plc) and type D (plc + etx), whereas non-significant connotation was observed in the case of type D (plc + β2 + etx). Type A (plc + β2) had maximum response and type A (plc), type D (plc + etx), and type D (plc + β2 + etx) were subsequently less responsive in contribution level (Figure 4).

From the PCA plot of both cattle and buffalo it can be assured that all the toxinotypes had significant variation.

### 2.4. Correlation among the Toxinotypes of C. perfringens in Buffalo

**Figure 5:**
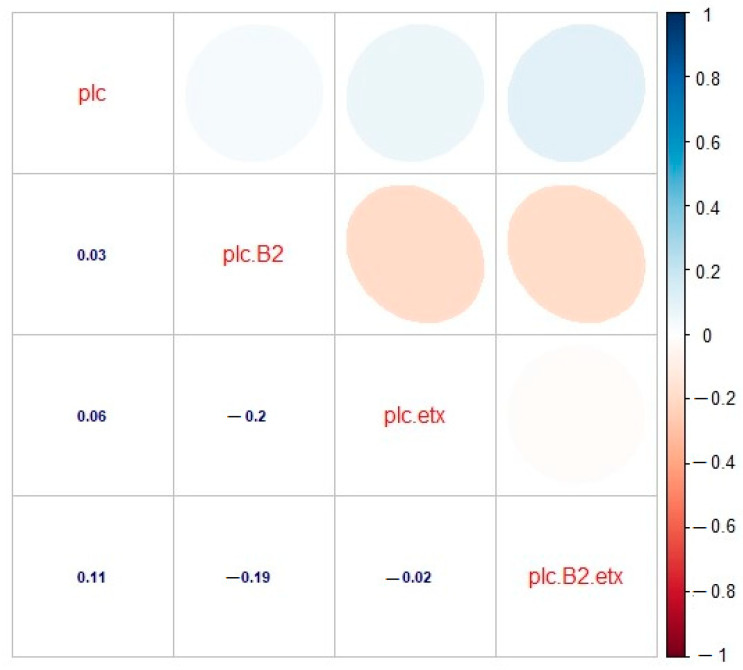
Correlation analysis for toxinotypes A (plc), A (plc + β2), D (plc + etx), and D (plc + β2 + etx) of *C. perfringens* in buffalo. plc: type A (plc), plc.B2: type A (plc + β2), plc.etx: type D (plc + etx), plc.B2.etx: type D (plc + β2 + etx). The digits in blue depict and the ellipses describe the r value (correlation value) of the tested toxinotypes.

**Figure 6:**
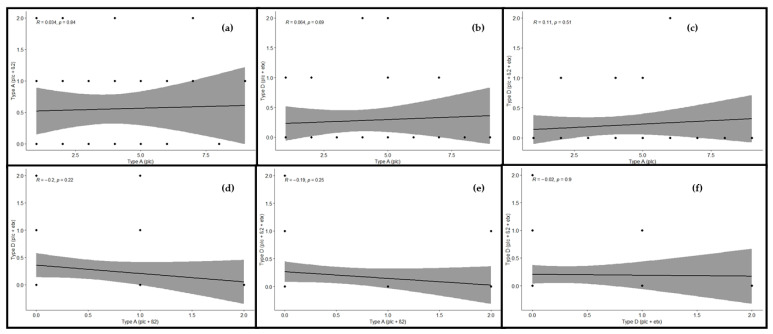
Relationship among toxinotypes of *C. perfringens* in buffalo using regression analysis. The regression line shows the extent of correlation among the genotypes. Regression analysis between (**a**) type A (plc) and type A (plc + β2), (**b**) type A (plc) and type D (plc + etx), (**c**) type A (plc) and type D (plc + β2 + etx), (**d**) type A (plc + β2) and type D (plc + etx), (**e**) type A (plc + β2) and type D (plc + β2 + etx), and (**f**) type D (plc + etx) and type D (plc + β2 + etx). “*R*” depicts the extent of correlation and “*p*” depicts the level of significance (*p* ≤ 0.05).

Correlation was estimated between various toxinotypes of *C. perfringens* in buffalo, and significant correlation was observed. A significant and positive correlation was observed between toxinotypes A (plc) and A (plc + β2) (r = 0.03), which revealed that type A (plc) and type A (plc + β2) showed slightly less correlation with each other. The correlation between genotype A (plc) and D (plc + etx) was also found to be positive and significant (r = 0.06), which revealed much less relationship among the correlated genotypes. As far as the correlation between type A (plc) and type D (plc + β2 + etx) was concerned, there existed an appropriate correlation between said genotypes. Observing these genotypes, a positive and significant correlation was observed (r = 0.11). Looking at genotype A (plc + β2) and D (plc + etx), significant and negative correlation was observed (r = –0.2). Correlation between type A (plc + β2) and type D (plc + β2 + etx) was also found to be negative and significant (r = –0.19), whereas correlation between toxinotypes D (plc + etx) and D (plc + β2 + etx) was found to be slightly negative and significant, as they were found to be inverse to each other (r = –0.02). Observing the correlation matrix among the toxin genotype clarified that the type A (plc) toxin genotype was positive and the correlations among type A (plc + β2), type D (plc + etx), and type D (plc + β2 + etx) was found to be negative and had their own significant impact (Figure 5). This relationship among the toxinotypes were also illustrated with the regression analysis, which also depicted the same response as the correlation matrix (Figure 6).

### 2.5. Correlation among the Toxinotypes of C. perfringens in Cattle

**Figure 7:**
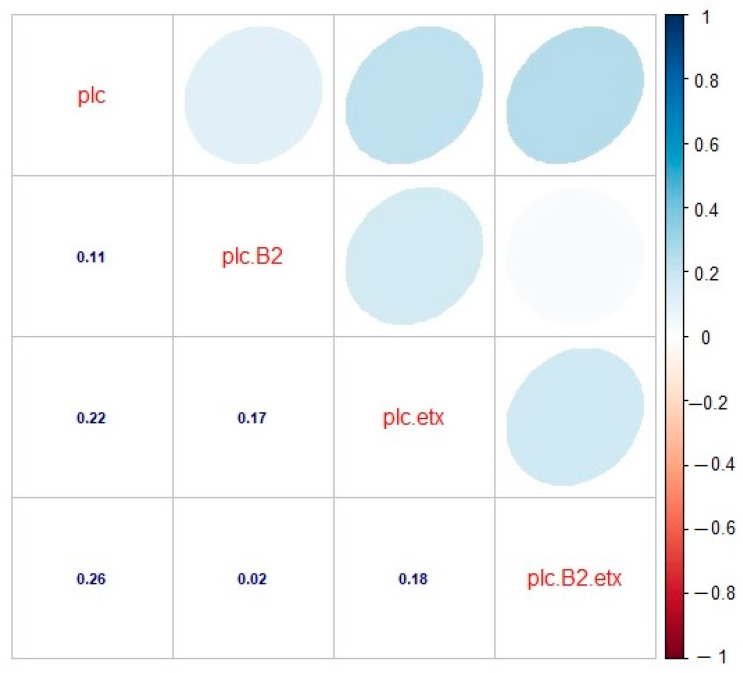
Correlation analysis for toxinotypes A (plc), A (plc + β2), D (plc + etx), and D (plc + β2 + etx) of *C. perfringens* in cattle. plc: type A (plc), plc.B2: type A (plc + β2), plc.etx: type D (plc + etx), plc.B2.etx: type D (plc + β2 + etx). The digits in blue depict and the ellipses describe the *r* value (correlation value) of the tested toxinotypes.

In cattle, the correlation among the toxinotypes was found to be significant. The correlation between genotypes A (plc) and A (plc + β2) was found to be significant and positive (r = 0.11), as there was some relation between them. The correlation was found to be positive and significant in the case of genotypes A (plc) and D (plc + etx), as they depicted a significant correlation between them (r = 0.22). There also existed a positive and significant correlation between genotypes A (plc) and D (plc + β2 + etx), as both genotypes showed a strong relationship with each other (r = 0.26). Looking at the correlation between genotypes A (plc + β2) and D (plc + etx), a positive and significant correlation (r = 0.17) was observed, which shows some sort of significance among these genotypes. The correlation between genotypes A (plc + β2) and D (plc + β2 + etx) was found to be positive and significant, as they had an adequate relationship with each other (r = 0.02), and the correlation between genotypes D (plc + etx) and D (plc + β2 + etx) was found to be positive (r = 0.18) and significant, as their relationship was substantial (Figure 7).

**Figure 8:**
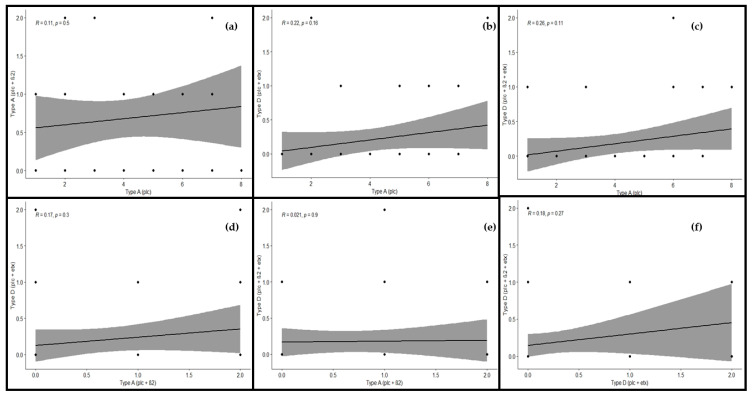
Relationship among toxinotypes of *C. perfringens* in cattle using regression analysis. The regression line shows the extent of correlation among the genotypes. Regression analysis between (**a**) type A (plc) and type A (plc + β2), (**b**) type A (plc) and type D (plc + etx), (**c**) type A (plc) and type D (plc + β2 + etx), (**d**) type A (plc + β2) and type D (plc + etx), (**e**) type A (plc + β2) and type D (plc + β2 + etx), and (**f**) type D (plc + etx) and type D (plc + β2 + etx). “*R*” depicts the extent of correlation and “*p*” depicts the level of significance (*p* < 0.05).

From the correlation matrix it was confirmed that all the toxinotypes exhibited significant and positive correlation with each other. This association among the toxinotypes was also illustrated in the regression analysis, which also depicted the same response as the correlation matrix (Figure 8).

### 2.6. Cluster Analysis of C. perfringens Toxinotypes in Buffalo and Cattle

**Figure 9:**
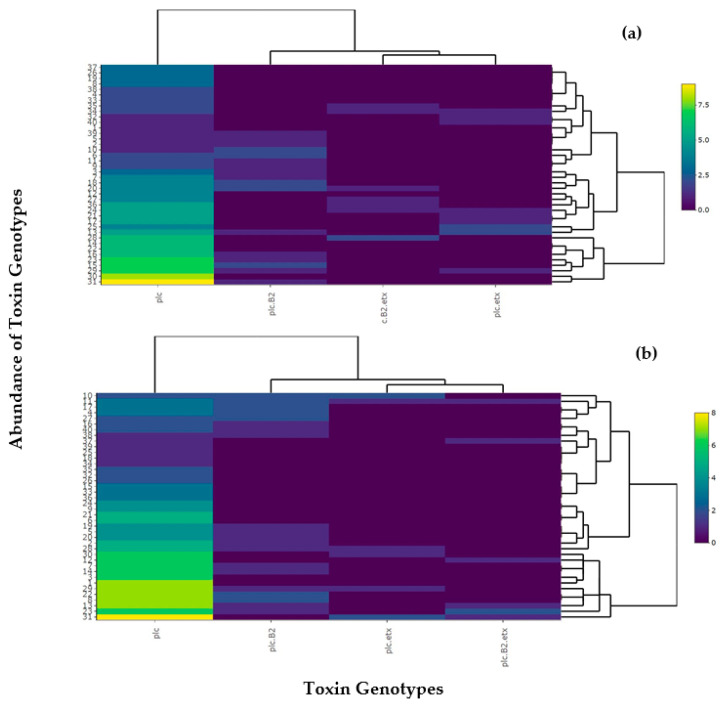
Visualization of heatmap for relative abundance of toxinotypes present in *C. perfringens* in (**a**) buffalo and (**b**) cattle. Different colors in the figure shows the abundance of toxinotypes and the gradient shows the availability level of the toxinotypes in the animals according to the scale mentioned on the right side.

For the determination of relative abundance of *C. perfringens* toxinotypes, a heatmap was developed that assessed the approximate amount of prevalent toxinotypes in buffalo and cattle. Type A (plc) was found to be more abundant among most buffalo and cattle. Type A (plc + β2) was found to be more abundant in both buffalo and cattle. Buffaloes with type D (plc + etx) and type D (plc + β2 + etx) were found to be less abundant compared to type A. It can be concluded that all toxinotypes showed appearance in both buffalo and cattle but type A (plc) showed maximum appearance among all the toxinotypes (Figure 9).

From the heatmap, toxin reactivity and interaction among the toxin was depicted and the prevalence of these toxinotypes was appropriately assessed per animal (buffalo and cattle).

### 2.7. Optimization of All Toxinotypes in C. perfringens of Buffalo and Cattle Using Response Surface Methodology

All the toxinotypes of *C. perfringens* that came under investigation were assessed based on response surface methodology via Box–Behnken design. The following quadratic response surface model was fitted to the data.

(1) $$Y=\beta_{0}+\overset{k}{\underset{i=1}{\sum }}\beta_{i}F_{i}+\overset{k}{\underset{i=1}{\sum }}\beta_{\mathrm{ii}}F_{i}^{2}+\sum \overset{k}{\underset{i<j=1}{\sum }}\beta_{\mathrm{ij}}F_{i}F_{j}+\varepsilon$$

where “Y” is the abundance of toxinotypes in buffalo and cattle, “β_0_” is the intercept constant, and “βi”, “βii”, and “βij” are the regression coefficients. “F_i_” and “F_j_” re coded values of the toxinotypes and “ε” is the error term. The distribution of coded variables to the toxintotypes is mentioned in Table 2.

For the optimization of toxinotypes, an analysis of variance was performed via quadratic model and the abundance of these genotypes was checked in buffalo and cattle. The following regression equation was obtained in buffalo:

Y = 2.791 + 0.417 A − 0.386 B − 0.255 C − 0.0033 D − 0.071 AB − 0.048 AC + 0.00 AD + 0.0875 BC − 0.0071 BD + 0.130 CD − 0.0011 A² + 0.0627 B² − 0.0798 C² + 0.0039 D²(2)

According to this regression equation, type A (plc) had a positive effect as well as increasing abundance whereas type A (plc + β2) showed a negative effect. Type D (plc + etx) and type D (plc + β2 + etx) also showed a negative effect. The interaction between all toxinotypes was found to be negative whereas the interaction of type D (plc + etx) with type D (plc + β2 + etx) showed positive impact. The squares of all toxin genotypes A (plc) and D (plc + etx) showed a negative impact and the remaining genotypes showed a positive impact.

According to analysis of variance (Table 3), the model fitted was found to be significant, as the *p*-value was less than 0.05. All the toxinotypes were also found to be significant (*p* < 0.05). The interaction of all the toxinotypes was also found to be significant except for type A (plc) and type D (plc + β2 + etx), which implies that all the toxinotypes showed substantial abundance in combinations as well. This reveals that *C. perfringens* has an appropriate abundance of genotypes that have high infectivity.

**Figure 10:**
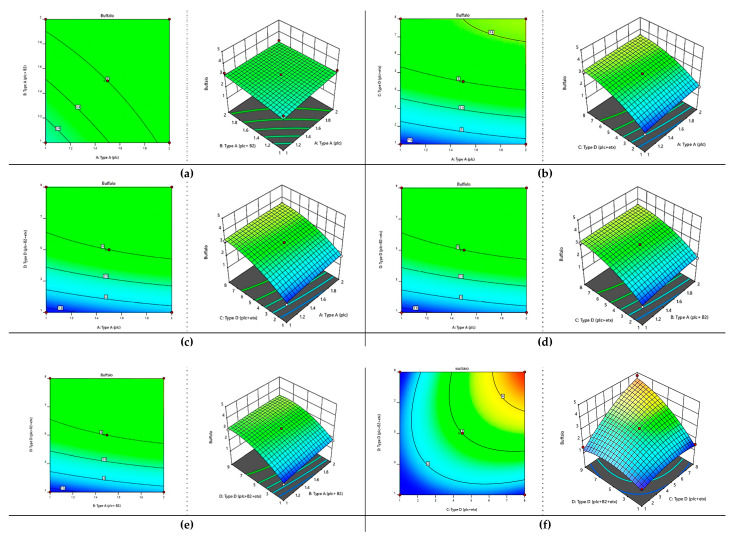
Optimization of toxinotypes of *C. perfringens* in buffalo via response surface methodology. Contour and surface plots of (**a**) type A (plc) with type A (plc+ β2), (**b**) type A (plc) and type D (plc + etx), (**c**) type A (plc) and type D (plc+ β2 + etx), (**d**) type A (plc+ β2) and type D (plc+ β2 + etx), (**e**) type A (plc+ β2) and type D (plc+ β2 + etx), and (**f**) type D (plc + etx) and type D (plc+ β2 + etx)**.**

Based on the analysis of variance applied to this model, it became obvious that this model showed significant response at the 5% level of significance and this model was also very suitable and reproducible due to having very little lack of fit (*p* > 0.05). Thus, the optimum parameters were also defined as shown in Table 3 and Figure 10. The contour and surface plots developed based upon this analysis also show that *C. perfringens* has a substantial amount of toxinotypes that can pose a huge effect on the health of buffalo.

According to the values mentioned in Table 4, it was concluded that in runs 5 and 11, the buffalo showed a maximum abundance of all four genotypes present in *C. perfringens*.

For the optimization of toxinotypes of *C. perfringens* in cattle, the following regression equation was found.

Y = 2.83 + 0.539 A − 0.586 B − 0.0768 C − 0.07685 D − 0.144 AB − 0.0208 AC − 0.0208 AD + 0.0431 BC + 0.0431 BD + 0.115 CD − 0.0135 A² + 0.1234 B² − 0.0741 C² − 0.0741 D²(3)

According to the regression equation, all the toxinotypes showed negative responses except type A (plc). The interaction of all toxinotypes was found to be significant but negative except for type A (plc + β2) with type D (plc + etx) and type A (plc + β2) and type D (plc + β2 + etx), which were found to be positive. This shows that all the toxinotypes showed significant abundance in combinations. This reveals that *C. perfringens* has an appropriate abundance of genotypes in cattle that also have high infectivity. The squares of all the genotypes responded negatively except for type A (plc + β2).

**Figure 11:**
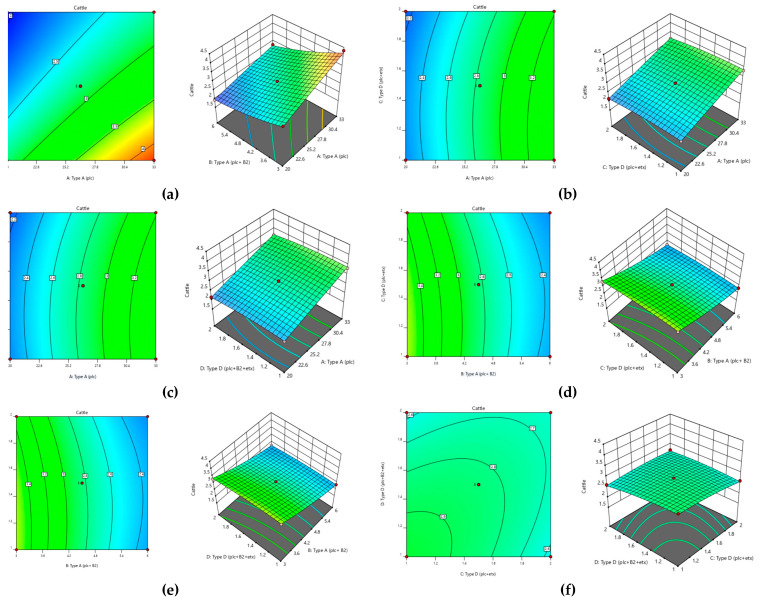
Optimization of toxinotypes of *C. perfringens* in cattle via response surface methodology. Contour and surface plots of (**a**) type A (plc) with type A (plc+ β2), (**b**) type A (plc) and type D (plc + etx), (**c**) type A (plc) and type D (plc+ β2 + etx), (**d**) type A (plc+ β2) and type D (plc+ β2 + etx), (**e**) type A (plc+ β2) and type D (plc+ β2 + etx), and (**f**) type D (plc + etx) and type D (plc+ β2 + etx)**.**

All the variables, along with their interaction and squares, were found to be significant. Based on the analysis of variance (Table 5) applied to this model, it became obvious that this model showed significant response at the 5% level of significance and this model was also very suitable and reproducible due to having very little lack of fit (*p* > 0.05). The coefficient of determination (R^2^) also confirmed that with 99% surety this data is highly significant and can be applied under various conditions. Thus, the optimum parameters were also defined as shown in Table 5. The contour and surface plots (Figure 11) developed based upon this analysis also depict that *C. perfringens* has substantial amount of toxinotypes that can pose a huge effect on the health of cattle. 

According to the values mentioned in Table 6, it was concluded that in runs 9, 26, and 27, cattle showed the maximum abundance of all four genotypes present in *C. perfringens.*

**Figure 12:**
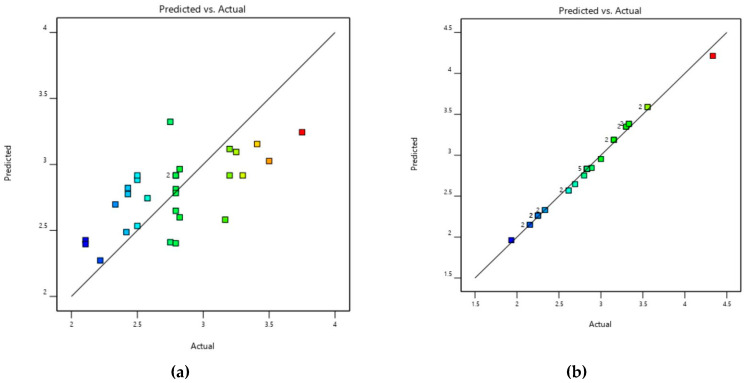
Validation of the model for (**a**) buffalo (**b**) cattle of *C. perfringens* toxinotypes in Punjab province, Pakistan.

Toxin genotypes were assessed using optimization by response surface methodology. All the applicable methods which can predict the occurrence of genotypes are presented in Table 7 and Figure 12.

## 3. Discussion

*C. perfringens* is a bacterium that has widespread occurrence and is capable of producing certain enzymes that have hydrolytic and toxic properties. Several pathovars of this bacterium evolve due to variable regions in chromosomes that encode toxin-producing genes. Each toxin-producing gene has certain genotypes that cause specific disease symptoms. For the purpose of pathovar identification, toxin genotyping has been found to be more consistent than classical toxinotyping [20].

Depending upon the pathovars of *C. perfringens* there are seven toxinotypes (A, B, C, D, E, F, and G) that mostly occur worldwide and produce six major toxins, i.e., α, β, ε, ι, *CPE*, and *NetB* [21]. Upon infection with the strain, these toxins cause toxicity in the host at an exponential growth rate [22,23,24]. Each toxin has its own specific properties and every toxin is accompanied with a certain disease either in humans or in animals [25]. The most commonly occurring genotype is type A, with widespread incidence as it causes human gangrene due to its α-toxin, θ-toxin, and several hydrolytic enzymes. Furthermore, it also exhibits an enterotoxin known as CPE, which causes gastrointestinal problems, particularly food poisoning [26]. Type A consists of the *cpe* gene, which causes food poisoning, fattening, and diarrhea. From the *cpe* gene, the identification of enterotoxigenic pathovars is possible [27,28]. Another novel toxin, β-2, has been identified, originally known as toxin type C, and is involved in necrotic enteritis. The strain can be effectively managed via vaccination [29,30].

The current study was aimed at sorting out the toxinotypes based on *C. perfringens* origins in cattle and buffalo in Punjab province of Pakistan. Out of all the toxinotypes (A–G), the most prevalent toxin genotypes were type A (plc) and type A (plc + β2), as most of the infected animals showed both of these genotypes at a maximum rate. In Central Punjab (Lahore, Sahiwal, and Cheecha Watni), 60% of the sampled buffalo and 73% of the sampled cattle and in Southern Punjab (Bhakkar, Dera Ghazi Khan, and Bahawalpur), 64% of sampled buffalo and 57% of the sampled cattle showed the type A (plc) genotype. In total, 149 buffalo out of 240 and 22 buffaloes out of 240 showed type A (plc + etx) and type A (plc + β2) genotypes, respectively, whereas 157 cattle out of 240 and 21 cattle out of 240 showed type A (plc) and type A (plc + β2) genotypes, respectively, in both Central and Southern Punjab. Other toxinotypes (B, C, E, F, and G) were not observed after PCR detection.

These results are in some agreement with Forti et al. (2020) [31], who examined 632 *C. perfrignens* isolates in Italy. Through PCR, it was observed that 93% of the total strains exhibited toxinotype A, type D was found in 3% isolates, type F was observed in 2.5%, and type B and type E were found much less. Type G was not found in any of the observed strains.

Marks et al. (2002) also described that *C. perfringens* showed type A and type D toxin forms in dogs. When *C. perfringens* infected the dogs there were two genotypes that were found responsible for causing diarrhea in them [32]. Moreover, Twedt (1993) [33] also reported that the strains of *C. perfringens* that produce the toxins can be primarily detected via diarrhea symptoms and feces of the infected host. *C. perfringens* shows 30–60% infection among various species of animals and birds [34,35].

During the study, the toxinotypes were analyzed according to their principal components in both buffalo and cattle. All the toxinotypes were found to be significantly associated with each other in buffalo and cattle. These findings show an association with Ferrarezi et al. (2008), Guran et al. (2013), and Yadav et al. (2017) [36,37,38] who observed that genotypes A and D showed some association in neonatal calves and chickens along with their related genes but that their role could be varied to a certain extent. In humans, these genotypes can cause gastrointestinal diseases, and infectivity has also been observed in milk-producing animals. Miwa et al. (1997) [39] and Haque et al. (2018) [40] confirmed that *C. perfringens* consists of the *cpe* gene, which is found in various isolates. This gene produces enterotoxin, which is the main cause of disease in human and animals. 

The relationship among the toxinotypes was then studied via correlation and regression analysis. These analyses confirmed that toxinotypes type A (plc) and type A (plc + β2) and type D (plc + etx) showed significant and negative correlation among each other, whereas type D (plc + β2 + etx) showed no correlation with other genotypes. These results are in some agreement with Athira et al. (2018) [41], who described the diversity of *C. perfringens* toxin genotypes in neonatal calves. Overall incidence of *C. perfringens* was found to be 37%. In this, 59% were type A and 37% were type C. Type B was around 15% and type E was 16%. Therefore, there was a diverse range of toxin genotypes in neonatal calves across Northern India.

Marks et al. (2002) [32] observed that both genotypes A and D showed some association with each other and that this association was found to be significant in causing diarrhea in dogs. 

The abundance of toxinotypes isolated from *C. perfringens* was also assessed via heatmap, which proved that all the genotypes showed significant occurrence in buffalo and cattle, as most of the animals showed all or some signs of toxinotypes. These results show some agreement with Netherwood et al. (1998), Møller et al. (1996), Yamagishi et al. (1997), Yoo et al. (1997), Daube et al. (1996), Fach et al. (1997), and Wieckowski et al. (1998) [27,42,43,44,45,46,47], who concluded that detection of the *cp*e gene determines the abundance of toxinotypes in *C. perfringens*. The genotyping technique is more accurate and helps in increasing the accuracy of determining toxinotypes.

For the optimization of all toxinotypes to determine the abundance in buffalo and cattle, the Box–Behnken design was used. It was observed that most of the buffalo and cattle showed the presence of type A and type D toxinotypes. These results show some resemblance with Riazi et al. (2015) [48], who reported that barberry extract and grape must work as natural preservatives against *C. perfringens.*

Response surface methodology was established to determine the fitness of a mathematical model and its graphical representation. It is broadly used in the field of chemometrics. This consists of a group of techniques that clarifies the fitness of the empirical model in a statistical design. To achieve the objective of optimization, linear, polynomial, or quadratic equations are used to properly describe the system address consolidated variables [49,50,51].

For the application of response surface methodology, it must be kept in mind that experimental variables and the experimental region should be properly defined as the number of variables determines the order of the model either factorial or quadratic. In response to surface methodology, many designs are used to determine the optimization of variables, including Box–Behnken, central composite, and Doehlert designs [52].

In response to the surface methodology, the selection of the variable that is affecting the system is most important because the study is based on it. The selection of an appropriate variable is also needed to study experimental effects and evaluate these effects, which have major importance in the experiment. Based on this fact these variables turn their interactions giving significant impacts in the system start, especially so that practical experiments become feasible [53]. The choice of experimental design is needed for proper description and explanation of variables in a system. Afterward, the mathematical and statistical analysis to obtain the fitness of the data is necessary to get an optimal region for all the treatments applied to gain an opposite result [54]. 

The Box–Behnken design suggests that the point is to be selected in an experiment with three-level factorial planning. This not only helps us with an efficient estimation of the coefficients in a model but also can be assisted for figuring a large number of variables [55,56].

The contour and surface plots developed after the analysis are required to achieve the best conditions. These plots help in the visual examination of the experiment under study. For quadratic models, the minimum–maximum and critical points are needed to determine the effectiveness of the mathematical function [57].

## 4. Conclusions

In Pakistan, the abundance of toxin genotypes exhibited by *C. perfringens* poses a huge threat to successful bovine farming, as type A and type D were found, with type A being the most abundant in both buffalo and cattle. For appropriate diagnosis, genotyping could be a beneficial approach to properly understanding the occurrence of the toxin genotypes as well as the toxin encoding genes responsible for enterotoxaemia.

## 5. Materials and Methods

### 5.1. Sample Collection

**Figure 13:**
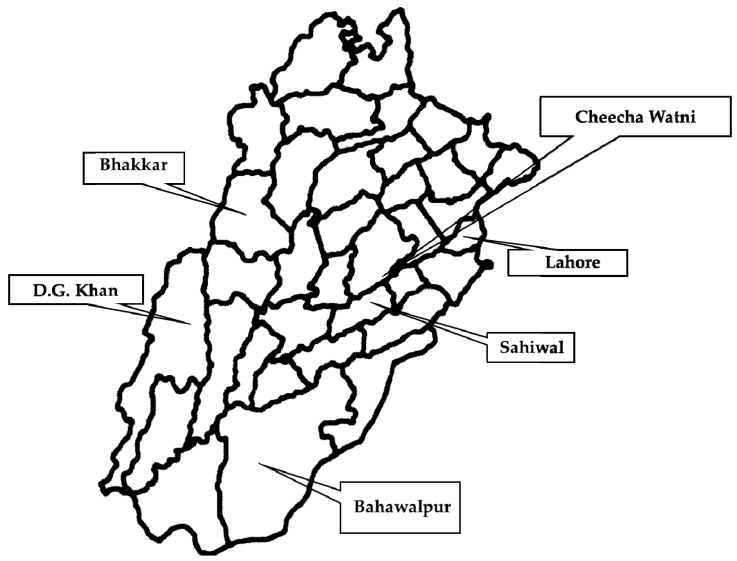
Sampling sites for collection and isolation of *C. perfringens*-based toxinotypes from buffalo and cattle in Punjab province of Pakistan. The areas with a black square denote the sampling sites from where samples for toxinotypes of *C. perfringens* were collected from various household and farm buffalo and cattle.

This study encompassed the major livestock sites in the Punjab province of Pakistan (Figure 12). The sampled areas were divided into two regions, i.e., Central Punjab and Southern Punjab. The cities where samples were collected in Central Punjab included Lahore (31.5204° N, 74.3587° E), Cheecha Watni (30.5373° N, 72.6983° E), and Sahiwal (30.6682° N, 73.1114° E) whereas in Southern Punjab, Bhakkar (31.6082° N, 71.0854° E), Dera Ghazi Khan (30.0489° N, 70.6455° E), and Bahawalpur (29.3544° N, 71.6911° E) were the cities where samples were collected. Intestinal contents of necropsied buffalo (n = 240) and cattle (n = 240) with a history of the intestinal problems were collected and transported to the laboratory for further processing (Figure 13). 

### 5.2. Isolation of Bacterial Strains

Fecal swabs were inoculated into 5 5mL thioglycolateate (FTA) broth and incubated at 37 ℃ (incubation chamber: Don Whitely DG-250 anaerobic workstation, United Kingdom) for 24 h. Subsequently, 100 μL of pre-enriched FTA broth was spread on tryptose sulphite cycloserine agar base enriched with 7% egg yolk and supplemented with D-cycloserine (Solarbio, Beijing, China). A black colony showing a positive lecithinase reaction was selected and cultured. For identification and purity of *C. perfringens*, they were streaked on Columbia blood agar (Huan Kai Microbial (HKM) Sci & Tech, Guangzhou, China) containing 5% defibrinated sheep blood and evaluated for typical double zone hemolysis associated with *C. perfringens*. Additionally, Gram staining and biochemical tests including glucose, maltose, an H_2_S reduction test, a nitrate reduction test, gelatin liquefaction, and a saccharose test, etc. (Hangwei, Microbiological Co Ltd., Hangzhou, China) were performed to confirm the identity of the *C. perfringens*. Isolates were preserved in 50% glycerol at −80°C until further use.

### 5.3. Extraction of Bacterial DNA

Genomic DNA of *C. perfringens* was extracted from overnight FTA broth culture inoculated with a single colony plate by Ultraclean Microbial DNA Isolation Kit (MoBio, Germantown, MD, USA), adhering to the manufacturer’s directives with minor modifications to attain high concentration. In brief, the bacterial suspension and lysis buffer mixed in an additional 20 μL of 20 mg/mL proteinase-K (Fisher Scientific Waltham, MA, USA). The reaction mixture was then incubated at 65 ℃ for 15 min to lyse the bacterial cell wall and to preclude DNase digestion to bacterial DNA. In order to prevent DNA degradation, a tube with a reaction mixture was placed on a flat pad horizontally and vortexed for 10 min. The serial dilution of DNA was performed with 25 μL 10 mM Tris- HCl (pH 8.0), and segregated on a 0.8% agarose gel (BD Biosciences, Franklin Lakes, NJ, USA). The quality assertion and purity of extracted DNA was determined by NanoDrop™ 2000 (Thermo Scientific Inc. Waltham, MA, USA). The absorbance ratio at 260 nm and 280 nm was employed to estimate protein contamination and the recommended purity level was at the A260/A280 ratio of 1.8 to 2.2. The DNA was stored at −20 ℃ for further genotyping analysis.

### 5.4. 16S rRNA Gene Sequence Analysis

The 16S rRNA gene was amplified with primer from the genomic DNA of each strain using 16S rRNA gene species-specific primer [21]. The sequences of the 16S rRNA gene region were determined by Sanger sequencing (Tsingke Biotechnology Company, Xian, China). The species were recognized by nucleotide alignments with the sequences of species banked in GenBank NCBI, USA (Bethesda MD, USA).

### 5.5. Assessment of Toxin Production by C. perfringens Isolates

Primers corresponding to the α toxin (cpa), β toxin (cpb), or ε toxin (ε-toxin) gene were used as described by Svensson et al. (2006) [58] (Table 8). Genes encoding toxin proteins were detected by PCR [21,59].

American Type Culture Collection ATCC-3624 (toxin type A, α-toxin positive) and China Institute of Veterinary Drug Control, Beijing, China, including CVCC-54 (toxin type B, α-, β- and ε-toxin positive), CVCC-61 (toxin type C, α-, and β-toxin positive) and CVCC-81 (toxin type D, α- and ε-toxin positive), were used as reference strains for toxinotyping (A, B, C, and D, respectively) and as positive controls for cpb2 and cpe. Amplification was performed in a 25 μL reaction mixture containing 50 ng template DNA, PCR Premix Taq (Ex Taq V.2.0 plus Dye Takara, Japan), and 0.5 μM of each primer on a thermocycler (Product: TaKaRa PCR Thermal Cycler DiceTM Touch Code TP350); the total reaction volume was adjusted with the addition of DNAase/RNAase (nuclease-free)-free water. The reaction conditions were as follows: initial denaturation at 96 ℃ for 5 min, 35 cycles of denaturation at 96 ℃ for 1 min., annealing at 56 ℃ for 1 min and elongation at 72 ℃ for 1 min, and a final extension at 72 ℃ for 10 min. 

For ε-toxin, the assay conditions were modified as follows: initial denaturation at 95 ℃ for 3 min, 35 cycles of denaturation at 95 ℃ for 40 s, annealing at 57 ℃ for 30 s and elongation at 72 ℃ for 30 s, and a final extension at 72 ℃ for 5 min. The amplified products were analyzed on a 1.2% agarose gel stained with ethidium bromide (10 mg/mL) by GenStar, Beijing, China. PCR-amplified products on the gels were extracted and purified with the E.Z.N.A^®^ Gel Extraction Kit (Omega Bio-Tek, Norcross, GA, USA) and sequenced (Tsingke Biotechnology Company, Xian, China) to ensure the identity with reference sequences.

### 5.6. Statistical Analysis

The data were subjected to statistical analysis using Statistical Package for Social Sciences (SPSS) version 26. ANOVA was performed and the means of the toxinotypes were compared using Tukey’s honestly significant difference (HSD) test. Principal component analysis (PCA), correlation, regression, and heatmaps among the toxinotypes were analyzed using R studio version 3.6.2. Optimization of all the genotypes was performed using response surface methodology via Box–Behnken design in Design Expert software version 12.

## Figures and Tables

**Table 1:** Frequency and percentage of *C. perfringens* toxinotypes A (plc), A (plc + β2), D (plc + etx), and D (plc + β2 + etx) isolated from buffalo and cattle in sampled areas of Central and Southern Punjab province in Pakistan.

Sampling Areas	Buffalo								Cattle							
	Type A (plc)		Type A (plc + β2)		Type D (plc + etx)		Type D (plc + β2 + etx)		Type A (plc)		Type A (plc + β2)		Type D (plc + etx)		Type D (plc + β2 + etx)	
	Central Punjab															
	Frequency	%age	Frequency	%age	Frequency	%age	Frequency	%age	Frequency	%age	Frequency	%age	Frequency	%age	Frequency	%age
Lahore	22/40	55	3/40	7.5	1/40	2.5	1/40	2.5	27/40	67.5	3/40	7.5	1/40	2.5	1/40	2.5
Sahiwal	23/40	57.5	4/40	10	2/40	5	1/40	2.5	28/40	70	6/40	15	1/40	2.5	1/40	2.5
Cheecha Watni	27/40	67.5	5/40	12.5	2/40	5	2/40	5	33/40	82.5	5/40	12.5	1/40	2.5	2/40	5
Total	72/120	60%	12/120	10%	5/120	4.17%	4/120	3.33%	88/120	73.33%	14/120	11.67%	3/120	2.5%	4/120	3.33%
	**Southern Punjab**															
Bahawalpur	21/40	52.5	4/40	10	1/40	2.5	1/40	2.5	24/40	60	4/40	10	2/40	5	1/40	2.5
Bhakkar	25/40	62.5	3/40	7.5	3/40	7.5	2/40	5	25/40	62.5	6/40	15	2/40	5	1/40	2.5
D.G. Khan	31/40	77.5	3/40	7.5	2/40	5	1/40	2.5	20/40	50	3/40	7.5	1/40	2.5	1/40	2.5
Total	77/120	64.17%	10/120	8.33%	7/120	5%	4/120	3.33%	69/120	57.5%	13/120	10.83%	5/120	4.17%	3/120	2.5%
Grand Total	149/240	62.08%	22/240	9.17%	11/240	4.58%	8/240	3.33%	157/240	65.42%	21/240	11.25%	8/240	3.34%	7/240	2.92%

**Table 2:** A design approach for determining the optimization of toxinotypes of *C. perfringens* in buffalo and cattle via Box–Behnken design.

Toxinotypes	Coded Symbol	Range			
		Buffalo		Cattle	
		−1	+1	−1	+1
**Type A** (plc)	A	21	31	20	33
**Type A** (plc + β2)	B	3	5	3	6
**Type D** (plc + etx)	C	1	3	1	2
**Type D** (plc + β2 + etx)	D	1	2	1	2

**Table 3:** Analysis of variance of *C. perfringens*-based toxinotypes (type A (plc), type A (plc + β2), type D (plc + etx), and type D (plc + β2 + etx)) for a quadratic model in buffalo via Box–Behnken design.

Source	Sum of Squares	Degree of Freedom	Mean Square	F-Value	*p*-Value
**Model**	4.88	14	0.3483	91.86	<0.0001
A-Type A (plc)	2.09	1	2.09	550.39	<0.0001
B-Type A (plc + β2)	1.79	1	1.79	472.93	<0.0001
C-Type D (plc + etx)	0.7846	1	0.7846	206.93	<0.0001
D-Type D (plc + β2 + etx)	0.0001	1	0.0001	0.0360	0.0500
AB	0.0204	1	0.0204	5.38	0.0360
AC	0.0095	1	0.0095	2.50	0.0160
AD	0.0000	1	0.0000	0.0000	1.0000
BC	0.0307	1	0.0307	8.08	0.0130
BD	0.0002	1	0.0002	0.0538	0.0199
CD	0.0680	1	0.0680	17.93	0.0008
A²	9.264 × 10^−6^	1	9.264 × 10^−6^	0.0024	0.0313
B²	0.0255	1	0.0255	6.74	0.0212
C²	0.0414	1	0.0414	10.91	0.0052
D²	0.0001	1	0.0001	0.0263	0.0136
**Residual**	0.0531	14	0.0038		
Lack of Fit	0.0031	10	0.00031	0.0248	0.8371
Pure Error	0.05	4	0.0125		
**Cor Total**	4.93	28			

**Table 4:** Observed and predicted values of *C. perfringens* toxinotypes (type A (plc), type A (plc + β2), type D (plc + etx), type D (plc + β2 + etx)) for a quadratic model in buffalo obtained after optimization analysis from response surface methodology via Box–Behnken design.

Runs	Type A (plc)	Type A (plc + β2)	Type D (plc + etx)	Type D (plc + β2 + etx)	Buffalo (No.)	
					Observed Values	Predicted Values
1	21	4	1	2	3	2
2	26	4	2	2	3	2
3	21	5	2	2	2	1
4	21	3	2	2	3	2
5	26	3	1	2	4	3
6	21	4	2	2	2	1
7	26	4	2	2	3	2
8	31	4	1	2	3	2
9	31	5	2	2	3	2
10	26	5	2	2	3	1
11	31	3	2	2	4	3
12	26	5	2	1	2	1
13	26	5	3	2	2	1
14	31	4	2	1	3	2
15	26	3	2	1	3	2
16	26	3	2	2	3	2
17	26	4	2	2	3	2
18	26	5	1	2	3	2
19	26	4	2	2	3	2
20	26	4	2	2	3	2
21	21	4	3	2	2	1
22	21	4	2	1	2	1
23	31	4	2	2	3	2
24	26	4	1	1	3	2
25	26	4	3	1	2	1
26	26	4	3	2	3	2
27	26	3	3	2	3	2
28	31	4	3	2	3	2
29	26	4	1	2	3	2

**Table 5:** Analysis of variance of *C. perfringens*-based toxinotypes (type A (plc), type A (plc + β2), type D (plc + etx), type D (plc + β2 + etx)) for a quadratic model in cattle via Box–Behnken design.

Source	Sum of Squares	Degree of Freedom	Mean Square	F-Value	*p*-Value
**Model**	8.13	14	0.5804	190.91	<0.0001
A-Type A (plc)	3.50	1	3.50	1150.20	<0.0001
B-Type A (plc + β2)	4.12	1	4.12	1355.95	<0.0001
C-Type D (plc + etx)	0.0709	1	0.0709	23.31	0.0003
D-Type D (plc + β2 + etx)	0.0709	1	0.0709	23.31	0.0003
AB	0.0835	1	0.0835	27.45	0.0001
AC	0.0017	1	0.0017	0.5711	0.0424
AD	0.0017	1	0.0017	0.5711	0.0424
BC	0.0074	1	0.0074	2.44	0.0147
BD	0.0074	1	0.0074	2.44	0.0147
CD	0.0533	1	0.0533	17.52	0.0009
A²	0.0012	1	0.0012	0.3928	0.0149
B²	0.0988	1	0.0988	32.49	<0.0001
C²	0.0357	1	0.0357	11.73	0.0041
D²	0.0357	1	0.0357	11.73	0.0041
Residual	0.0426	14	0.0030		
Lack of Fit	0.0026	10	0.0026	0.26	0.8934
Pure Error	0.04	4	0.01		
**Cor Total**	8.17	28			

**Table 6:** Observed and predicted values of *C. perfringens* toxinotypes (type A (plc), type A (plc + β2), type D (plc + etx), and type D (plc + β2 + etx)) for a quadratic model in cattle obtained after optimization analysis from response surface methodology via Box–Behnken design.

Runs	Type A (plc)	Type A (plc + β2)	Type D (plc + etx)	Type D (plc + β2 + etx)	Cattle	
					Observed Values	Predicted Values
1	33	6	2	2	3	2
2	27	3	2	2	3	2
3	27	5	2	2	3	2
4	33	5	1	2	3	2
5	20	3	2	2	3	2
6	27	5	2	1	3	2
7	27	5	2	2	3	2
8	20	5	2	2	2	1
9	33	3	2	2	4	3
10	27	6	2	1	2	1
11	27	5	2	2	3	2
12	20	5	2	2	2	1
13	27	5	2	2	3	2
14	20	5	2	1	2	1
15	20	6	2	2	2	1
16	27	6	2	2	2	1
17	33	5	2	2	3	2
18	27	5	2	2	3	2
19	27	5	1	1	3	2
20	27	3	2	2	3	2
21	27	5	1	2	3	2
22	33	5	2	1	3	2
23	27	6	2	2	2	1
24	20	5	1	2	2	1
25	27	6	1	2	2	1
26	27	3	2	1	4	3
27	27	3	1	2	4	3
28	27	5	2	2	3	2
29	33	5	2	2	3	2

**Table 7:** Summary statistics for responses used in the models.

Source	R^2^	Adjusted R^2^	Predicted R^2^	Remarks
Model summary statistics for isolated toxinotypes (A and D) in Buffalo				
Linear	0.1330	0.0115	0.3067	
2-Factor Interaction	0.2280	0.2008	0.3266	
Quadratic	0.9842	0.8415	0.9695	Suggested
Cubic	0.8872	0.8262	0.9122	Aliased
Model summary statistics for isolated toxinotypes (A and D) in Cattle				
Linear	0.9501	0.9418	0.9219	
2-Factor Interaction	0.9691	0.9519	0.8978	
Quadratic	0.9948	0.9896	0.9700	Suggested
Cubic	0.9969	0.9857	0.5577	Aliased

**Table 8:** Primers used for the detection of *C. perfringens* toxin genes

Genes	Primer Sequences (5′–3′)	Size (bp)	References
plc	GCTAATGTTACTGCCGTTGACC CCTCTGATACATCGTGTAAG	324	(Meer and Songer, 1997)
β-2 toxin/Cbp-2	GAAAGGTAATGGAGAATTATCTTAATGC GCAGAATCAGGATTTTGACCATATACC	567	(Meer and Songer, 1997)
cpb	GCGAATATGCTGAATCATCTA GCAGGAACATTAGTATATCTTC	196	(Meer and Songer, 1997)
etx	CCACTTACTTGTCCTACTAAC GCGGTGATATCCATCTATTC	656	(Meer and Songer, 1997)
cpe	GGAGATGGTTGGATATTAGG GGACCAGCAGTTGTAGATA	233	(Meer and Songer, 1997)
iap	GGAAAAGAAAATTATAGTGATTGG CCTGCATAACCTGGAATGGC	461	(Rood et al., 2018)
netB	CTTCTAGTGATACCGCTTCAC CGTTATATTCACTTGTTGACGAAAG	738	(Rood et al., 2018)

## Data Availability

Not applicable.
